# Safety of Short-Term Supplementation with Methylliberine (Dynamine^®^) Alone and in Combination with TeaCrine^®^ in Young Adults

**DOI:** 10.3390/nu12030654

**Published:** 2020-02-28

**Authors:** Trisha A. VanDusseldorp, Matthew T. Stratton, Alyssa R. Bailly, Alyssa J. Holmes, Michaela G. Alesi, Yuri Feito, Gerald T. Mangine, Garrett M. Hester, Tiffany A. Esmat, Megan Barcala, Karleena R. Tuggle, Michael Snyder, Andrew S. Modjeski

**Affiliations:** 1 Department of Exercise Science and Sport Management, Kennesaw State University, Kennesaw, GA 30144, USA; abailly@students.kennesaw.edu (A.R.B.); alyssajh@hotmail.com (A.J.H.); malesi@students.kennesaw.edu (M.G.A.); yfeito@kennesaw.edu (Y.F.); gmangine@kennesaw.edu (G.T.M.); ghester4@kennesaw.edu (G.M.H.); tesmat@kennesaw.edu (T.A.E.); megan.barcala@choa.org (M.B.); snyderme@mac.com (M.S.); modjeskia@gmail.com (A.S.M.); 2 Kinesiology and Sport Management, Texas Tech University, Lubbock, TX 79409, USA; matthew.stratton@ttu.edu; 3 Southern Regional Physician Management Group, LLC, Riverdale, GA 30274, USA; aneelrak@hotmail.com

**Keywords:** TeaCrine^®^, Dynamine^®^, theacrine, methylliberine, purine alkaloid

## Abstract

Methylliberine (Dynamine^®^; DYM) and theacrine (Teacrine^®^; TCR) are purine alkaloids purported to have similar neuro-energetic effects as caffeine. There are no published human safety data on DYM, and research on TCR is limited. The purpose of this study was to examine the effect of four weeks of DYM supplementation with and without TCR on cardiovascular function and blood biomarkers. One-hundred twenty-five men and women (mean age 23.0 yrs, height 169.7 cm, body mass 72.1 kg; *n* = 25/group) were randomly assigned to one of five groups: low-dose DYM (100 mg), high-dose DYM (150 mg), low-dose DYM with TCR (100 mg + 50 mg), high-dose DYM with TCR (150 mg + 25 mg), and placebo. Regardless of group and sex, significant main effects for time were noted for heart rate, systolic blood pressure, and QTc (*p* < 0.001), high-density lipoproteins (*p* = 0.002), mean corpuscular hemoglobin (*p* = 0.018), basophils (*p* = 0.006), absolute eosinophils (*p* = 0.010), creatinine (*p* = 0.004), estimated glomerular filtration rate (*p* = 0.037), chloride (*p* = 0.030), carbon dioxide (*p* = 0.023), bilirubin (*p* = 0.027), and alanine aminotransferase (*p* = 0.043), among others. While small changes were found in some cardiovascular and blood biomarkers, no clinically significant changes occurred. This suggests that DYM alone or in combination with TCR consumed at the dosages used in this study does not appear to negatively affect markers of health over four weeks of continuous use.

## 1. Introduction

Caffeine is the most widely consumed alkaloid in the world. Its acute, positive impact on concentration, mood, alertness, fatigue, pain/soreness perception, and exercise performance was well studied (for reviews, please see References [1,2]). Chronic, regular caffeine consumption may blunt the subsequent physiological effects of supplementation, likely through an increase in adenosine receptor concentration [3,4], thereby reducing caffeine’s stimulatory effects [5]. Furthermore, high dosages of caffeine may cause anxiousness and adverse neuroendocrine and cardiovascular effects [6]. As such, there is a great deal of interest in caffeine alternatives that may promote similar ergogenic outcomes, without these undesirable side effects. 

In addition to caffeine (1,3,7-trimethylxanthine), the purine alkaloids theacrine (1,3,7,9-tetramethyluric acid) and methylliberine (*O*(2),1,7,9-tetramethylurate) were identified in the seeds and leaves of various *Coffea* species [7,8]. This includes *Coffea arabic* and *Coffea canephora.* Theacrine was also been found in *Coffea liberica, Coffea dewevrei, Coffea abeokuta* [8,9,10], and kucha tea (*C. assamica* var. *kucha* (green tea)). Initially, caffeine accumulates in young leaves but it is gradually replaced by theacrine and liberine. Due to purported synthesis from caffeine in some plants and structural similarities to caffeine [5,11], both theacrine and methylliberine are theorized to produce similar physiological effects with less side effects, potentially due to their different affinities with adenosine receptors [11,12]. Although theacrine was first discovered in 1937 [13], little research (to our knowledge, only seven studies) was conducted on theacrine’s effects on human health and performance until recently [14,15,16,17,18,19,20]. Previous cell and animal model investigations on theacrine reported improved antioxidant capacity [20] and anti-inflammatory responses [21], as well as analgesic [21], antidepressive [22], and sedative/hypnotic [23] mood states. Feduccia and colleagues also demonstrated that theacrine serves as an adenosine receptor antagonist and enhances locomotion, likely attributed to theacrine’s effects not only on adenosine receptors, but also the dopaminergic systems [24]. Interestingly, despite theacrines’s similarities with caffeine, Feduccia et al. (2012) found that seven days of 48 mg/kg theacrine intraperitoneal injections did not induce sensitization or negatively affect tolerance [24], which is typical with chronic caffeine consumption. In contrast, less in known about methylliberine, which is hypothesized to have similar physiological properties as caffeine and theacrine. To date, the only study on methylliberine examined the effects of a >98.0% pure supplement (Dynamine®, Compound Solutions, Inc., Carlsbad, CA; referred to as DYM), in Wistar rats [25]. Following a 90-day supplementation period, dose–response toxicology evaluation reported no toxicologically relevant clinical effects or effects on clinical hematological parameters [25]. Thus, both theacrine and methylliberine appear to be safe in animal models, but information regarding their effect in humans is limited.

In 2016, a human study was published on TeaCrine® (Compound Solutions, Inc., Carlsbad, CA; referred to as TCR), a commercially available, chemical equivalent bioactive version of theacrine. The report indicated that eight weeks of supplementing with either 200-mg or 300-mg doses of TCR did not negatively affect blood or hemodynamic measures associated with clinical safety, and positively affected cholesterol [17]. The authors suggest that TCR may be a viable “nutraceutical” alternative to cholesterol-lowering drugs, and, of late, a great deal of research on “nutraceuticals” and “functional foods” (e.g., zinc, quercetin, resveratrol, grape-seed extract, black/green tea) that may reduce the burden of dyslipidemia and cardiovascular disease exists (for reviews, please see References [26,27]). However, no human research was conducted to date on methylliberine. Therefore, we sought to investigate the safety of orally consumed, commercially available methylliberine in humans, as well as expand on the limited information on theacrine by examining its combination with methylliberine. Specifically, the purpose of this study was to examine the effect of four weeks of DYM supplementation with and without TCR on parameters of cardiovascular function and blood biomarkers associated with health. As methylliberine was never studied in humans, a four-week time period was selected based on previous studies on novel dietary supplements [28,29,30]. Based on previous findings [14,17,25], we hypothesized that DYM alone or with TCR would not produce significant abnormal changes in cardiovascular function parameters of heart rate, blood pressure, or electrical conduction of the heart assessed via an electrocardiogram, nor hematological markers of health for both men and women. 

## 2. Materials and Methods 

### 2.1. Experimental Design

**Figure 1:**
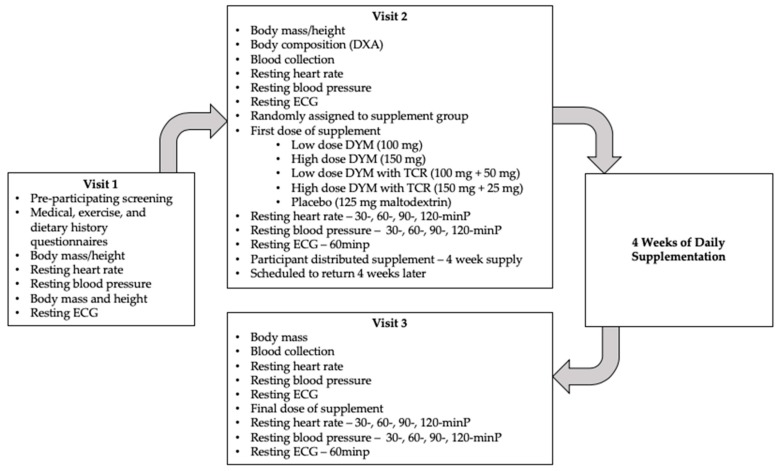
A flow chart depicting the overall study design. In total, 125 men and women were enrolled into the research study (60 men, 65 women). Following enrollment and screening for inclusion/exclusion criteria, participants consumed their randomly assigned supplement for four weeks. daily. Pre- and post-assessments of cardiovascular function (resting heart rate, blood pressure, and heart function via electrocardiogram) and blood collection for the analysis of complete blood count, comprehensive metabolic panel, and lipid panel were included. minP = minutes post first dose of randomly assigned supplement; DYM = Dynamine^®^; TCR = Teacrine^®^; mg = milligram.

Apparently healthy males and females took part in this placebo-controlled, double-blind investigation. Participants visited the laboratory fasted on three occasions: pre-participation screening (Visit 1), Visit 2, and Visit 3. Visit 2 and Visit 3 were separated by four weeks of once-daily supplementation. Participants were randomly assigned to one of five groups: low-dose DYM (100 mg), high-dose DYM (150 mg), low-dose DYM with TCR (100 mg + 50 mg), high-dose DYM with TCR (150 mg + 25 mg), and 125 mg of maltodextrin (placebo). Pre- and post-supplementation assessments included measures of cardiovascular function (i.e., resting heart rate, resting blood pressure, resting electrocardiogram (ECG)) and blood collection for the analysis of complete blood count (CBC), complete metabolic panel (CMP), and lipid panel. Prior to all visits to the laboratory, participants were asked to arrive in a fasted state (≥8 h), as well as abstain from caffeine (≥24 h) and exercise (≥24 h). The study was approved by the Kennesaw State University (KSU) Institutional Review Board (IRB #18-223). All data were collected in accordance with the Declaration of Helsinki. An overview of the study design may be seen in Figure 1.

### 2.2. Participants

Healthy weight to obese (not severely obese) men and women, 18–55 years, whom reported activity levels ranging from sedentary to recreationally active, were recruited for the study (no elite athletes) via flyers and word of mouth. Participants who reported a history of arrhythmias, a family history of sudden cardiac deaths in immediate family members (before the age of 55 for male, before the age of 65 for female relatives), and sensitivities to caffeine (anxiousness, nausea) were not enrolled. Participants diagnosed with or currently being treated for bacterial infections were excluded. Furthermore, participants were excluded if they were previously diagnosed with cardiovascular, endocrine/metabolic, gastrointestinal, renal, pulmonary, orthopedic, immunological, physiological, or musculoskeletal disorders. Individuals consuming more than 400 mg of caffeine per day, current smokers, or individuals who smoked within the last three years, as well as persons with known conditions or taking medications that may interfere with the absorption, distribution, metabolism, or excretion of purine alkaloids, also did not partake. Anti-inflammatory agents, including corticosteroids and non-steroidal anti-inflammatory drugs (NSAIDS), were also not permitted. Furthermore, any participant that had a positive, verbal pre-study drug screening for the use of alcohol, tetrahydrocannabinol (THC)/cannabinoids, amphetamines, benzodiazepines, cocaine, opioids, phencyclidine, barbiturates, and cotinine was excluded. Any participant who missed three or more doses during the four-week supplementation period was considered non-compliant and was excluded from the analyses. Following an explanation of all study procedures risks and benefits, 161 men and women signed an KSU Institutional Review Board approved consent form to complete the study (#18-223). Out of all enrolled participants, 125 men (*n* = 60) and women (*n* = 65) completed the study (23.0 ± 3.3 years, 169.7 ± 8.9 cm, 72.1 ± 13.7 kg, 25.7% ± 8.4% body fat) (Table 1). Participant reasons for not completing the study were as follows: (1) participants were classified as hypertensive (stage two hypertension) per the 2017 American Heart Association Guidelines at Visit 1 (*n* = 8); (2) participants presented with an ECG abnormality at Visit 1 (*n* = 7); (3) participants missed three or more days of supplementation and were asked to not return for Visit 3 (*n* = 11); (4) participants did not return for Visit 3 due to schedule conflicts (*n* = 5); (5) participants self-reported illness during the supplementation period (*n* = 3; self-reported flu-like symptoms including chills, sweats, muscle aches); (5) participants began supplementation and stopped responding to follow-up contact for Visit 3 (*n* = 2). Men and women completing the study were equally distributed within each of the five groups (12 men, 13 women, *n* = 25 per group). All participants were asked to maintain their habitual dietary intake (i.e., make no changes other than start taking their assigned supplement when instructed) and exercise habits (i.e., if they started the study reporting sedentary, they were asked to maintain that level of activity; if they started the study recreationally active, they were asked to maintain that level of activity) for the duration of the study. Following study completion, participants were compensated with a gift card.

### 2.3. Visits Overview

#### 2.3.1. Visit 1: Pre-participation screening 

Prior to starting the experimental portion of the study, all participants visited the laboratory for pre-participation screening (Visit 1). Each individual was firstly given an overview of the investigation, then provided their written informed consent to participate, and finally completed medical, exercise, and dietary questionnaires. Following completion of study documents, participants were asked to rest for 5 min in the seated position before completing resting heart rate and blood pressure assessments (two measurements separated by 2 min). If the two measurements varied by more than ±2 mmHg, a third measurement was taken and the average of the closest two values was used for later analysis. Subsequently, height and body mass were collected and then participants were prepared for and completed baseline, resting ECG assessment by a trained member of the research team. The ECG printout was reviewed by a medical doctor or clinical exercise physiologist for abnormalities. Qualifying participants were scheduled for Visit 2. 

#### 2.3.2. Visits 2 and 3 

Visit 2 began with assessments of body mass and composition (dual energy X-ray absorptiometry (DXA)), followed by blood collection, resting blood pressure, and heart rate, and lastly, a resting ECG. A blinded research team member then randomly assigned the individual to a study group. Participants then consumed their first dose and remain in the laboratory for 2 h. Resting heart rate and blood pressure were assessed every 30 min post (minP) first dose supplementation (30 minP, 60 minP, 90 minP, and 120 minP), while resting ECG was assessed at 60 minP only. Following the 120-minP time point, participants were given a bottle of their assigned supplement (coded for double-blind administration), instructed on how to consume the supplement once daily, and scheduled to return ( ±2 h time frame from Visit 2) to the laboratory four weeks later for Visit 3. Visit 3 procedures occurred in the same manner as Visit 2, excluding the height assessment and DXA scan. 

### 2.4. Laboratory Assessments 

#### 2.4.1. Anthropometric Measurements

Body mass was measured at the beginning of each laboratory visit. Height was measured following enrollment into the investigation during the first visit only. Participants completed all mass and height measurements without shoes using the same calibrated stadiometer and scale (Tanita WB 3000, Arlington Heights, IL). For demographic purposes, participant’s baseline body fat percentage was assessed via a full body DXA scan (Lunar iDXA, General Electric, Chicago, IL). The DXA scanner was calibrated according to manufacturer guidelines and the positioning of the participant was conducted according to manufacturer recommendations. All assessments occurred in the fasted state (≥8 h).

#### 2.4.2. Blood sampling and analysis

Participant’s fasting venous blood was collected during Visits 2 and 3 from a vein in the antecubital space by a research team member trained in phlebotomy. Blood was collected into serum separator (SST) and ethylenediaminetetraacetic acid (EDTA) treated Vacutainer® tubes. SST tubes were allowed to clot for 10 min prior to centrifugation at room temperature for 15 min (Model #642E, Drucker Diagnostics, Port Matila, PA, USA). EDTA whole-blood samples were inverted 8–10 times immediately after collection. Whole blood and serum were analyzed by a third-party laboratory (Laboratory Corporation of America™) for CBC with differential, CMP, and lipids. The CBC with differential allowed for the quantification of white blood cells, red blood cells, hemoglobin, hematocrit, mean corpuscular volume, mean corpuscular hemoglobin, mean corpuscular hemoglobin concentration, red cell distribution width, platelets, neutrophils, lymphocytes, monocytes, eosinophils, basophils, absolute neutrophils (derived by multiplying the white blood cell count (WBC) by the percent of neutrophils in the differential WBC count; the percentage of neutrophils consists of the segmented/fully mature neutrophils plus the bands/almost mature neutrophils), absolute lymphocytes (absolute lymphocyte count = WBC times lymphocytes divided by 100), absolute monocytes (absolute monocyte count = WBC times percent monocytes times 100), absolute eosinophils (absolute eosinophils = WBC times eosinophils divided by 100), absolute basophils (calculated by multiplying the percentage of basophils by the total number of WBC), and immature granulocytes and absolute immature granulocytes (metamyelocytes, myelocytes; no bands or blast cells). The CMP allowed for the assessment of glucose, blood urea nitrogen, creatinine, estimated glomerular filtration rate, blood urea nitrogen–creatinine ratio, sodium, potassium, chloride, total carbon dioxide, calcium, total protein, albumin (A), total globulin (G), A/G ratio, total bilirubin, alkaline phosphatase, aspartate aminotransferase, and alanine aminotransferase. The lipid panel assessment consisted of total cholesterol, triglycerides, high-density lipoproteins, very-low density lipoproteins, and low-density lipoproteins. 

#### 2.4.3. Resting heart rate and blood pressure

Resting heart rate(s) and blood pressure(s) were collected during all three visits. Prior to the measurements of heart rate and blood pressure, participants were fitted with a heart rate monitor (T31 Coded Transmitter and Polar Pt1 Heart Rate Monitor, Polar® Electro Inc., Bethpage, NY) and asked to sit on a chair for five minutes with back support to allow the blood pressure to adjust to resting conditions. Systolic and diastolic blood pressures were assessed by trained members of the research team, manually, using a sphygmomanometer (Diagnostix™ 788, American Diagnostic Corporation, Hauppauge, NY, USA), cuff of the appropriate size (Adcuff™, American Diagnostic Corporation, Hauppauge, NY, USA), and a stethoscope (Classic II, 3M™ Littmann®, St. Paul, MN, USA). During PRE assessments (i.e. before visit supplementation), blood pressure was measured twice with a 2-min interval between the measurements and the average score of the two measures being recorded nearest to one mmHg. During 30-minP, 60-minP, 90-minP, and 120-minP assessments, only a single blood pressure was collected. Blood pressure was always collected in the same arm of each participant. 

#### 2.4.4. Resting Electrocardiogram

Resting ECG(s) were collected during all three visits. A 12-lead ECG was conducted (I–III, V1–V6, aVR, aVL, aVF) following proper skin prep procedures. All females were asked to wear a bra with no metal. Participants were asked to remain in the seated position with their feet flat on the ground for 10 min. Cardiac rhythm was monitored and recorded via a GE Case® 8000 from minutes 5–10. Data were printed from the 12-lead ECG machine and PR interval, QRS duration, corrected QTc, P axis, R axis, T axis, P duration, RR interval, and PP interval were recorded.

Reliability statistics were calculated from the pre-ECG recordings collected on the first two visits (i.e., prior to any supplementation) from a random sample of 10 men (23.9 ± 3.9 years; 178 ± 6 cm; 82.8 ± 10.5 kg; body mass index = 26 ± 2.2) and 10 women (24.4 ± 7.1 years; 164 ± 5 cm; 66.6 ± 19.1 kg; BMI = 25.9 ± 4.9) that were drawn from the entire sample with each group being represented. After confirming that no statistical differences existed between time points, intraclass correlation coefficients (ICC_3,1_) and standard error of the measurement (SEM) were calculated for PR interval (ICC_3,1_ = 0.85, SEM = 8.63 ms), QRS duration (ICC_3,1_ = 0.92, SEM = 3.52 ms), QT_C_ (ICC_3,1_ = 0.74, SEM = 16.9 ms), P axis (ICC_3,1_ = 0.84, SEM = 8.68°), R axis (ICC_3,1_ = 0.91, SEM = 7.00°), T axis (ICC_3,1_ = 0.79, SEM = 8.81°), P duration (ICC_3,1_ = 0.9, SEM = 4.84 ms), RR interval (ICC_3,1_ = 0.84, SEM = 96.8 ms), and PP interval (ICC_3,1_ = 0.9, SEM = 92.0 ms).

#### 2.4.5. Supplementation intervention

Participants were matched according to sex and then randomly assigned to one of five groups: low-dose DYM (100 mg), high-dose DYM (150 mg), low-dose DYM with TCR (100 mg + 50 mg), high-dose DYM with TCR (150 mg + 25 mg), and 125 mg of maltodextrin (placebo). The terms high- and low-dose are terms used specifically to differentiate between groups in this manuscript and should not be deemed as definitive “high” and “low” dosages for the given supplements. Participants were asked to take their supplement in the morning with water (~12 fluid ounces) and food. Supplement compliance was monitored by asking the participant to return their pill bottle with any pills missed upon arrival to a member of the research team. Research team members also verbally confirmed the number of pills missed with each participant to account for supplement compliance. All supplements were provided by Compound Solutions, Inc. Supplements were indistinguishable to participants, as the pills were the same color, shape, and texture. Supplements were distributed by members of the research team who were blinded to treatments. The supplement key for each group was kept by a member of the research team not involved in data collection. 

#### 2.4.6. Diet Tracking 

During the four-week trial, participants were asked to track their diet two weekdays and one weekend day, for a total of three days per week. Participants either recorded all food and drinks on a hard-copy diet log or into MyFitnessPal (Under Armour, Inc, Baltimore, MD). A single member of the research team entered any hard-copy diet logs into MyFitnessPal. Carbohydrate, fat, protein, and total calories were assessed from week one and week four to determine if participants maintained their normal diets. Dietary intakes for macronutrients and total calories are displayed in Table 8. 

### 2.5. Statistical Analysis

A priori analysis using a moderate effect size f (0.25), standard alpha (0.05), and maximum beta (0.95) for a five-group, four-time-point repeated measures design, statistical software (G*Power, v.3.1.9.4, Universitat Dusseldorf, Germany) indicated that a minimum of 60 total participants were necessary to observe statistical differences. Prior to group comparisons, the assumption of normality was verified by the Shapiro–Wilk test. Subsequently, separate three-way (group × sex × time) analyses of variance with repeated measures were performed on all measures of cardiovascular function and blood biomarkers. The Greenhouse–Geisser adjustment was applied when the assumption of sphericity was violated. Following any significant F-ratio, pairwise comparisons were performed using the Bonferonni post hoc analysis. The magnitude of significant differences was assessed by effect sizes (i.e., eta squared, ɳ^2^) and qualitatively described as small (0.2–0.49), moderate (0.5–079), or large (≥ 0.80) [31]. Dependent *t*-tests were used to determine differences in diet from week one to week four. Criterion alpha was set at *p* ≤ 0.05. All statistical analyses were performed using JASP 0.10.2 (Amsterdam, the Netherlands). All data are reported as means ± standard deviation. 

## 3. Results

### 3.1. Cardiovascular Function

**Figure 2:**
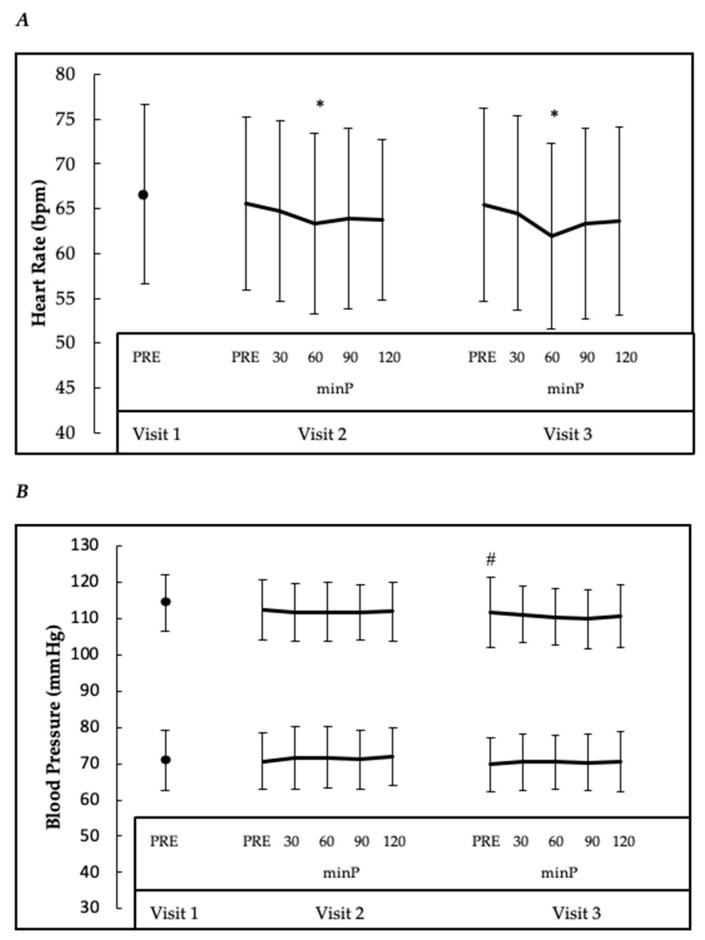
Collapsed data (*n* = 125; group and sex) for group changes in (**A**) heart rate and (**B**) blood pressure (systolic (toward the top) and diastolic (toward the bottom)). * Significantly (*p* < 0.05) different from respective visit’s PRE; # Different (*p* < 0.10) from Visit 1 (V1)*;* minP = minutes post PRE; mmHg = millimeters of mercury; bpm = beats per minute.

Regardless of group and sex, significant main effects for time were noted for heart rate (F = 6.87, *p* < 0.001, ɳ^2^ = 0.02), systolic blood pressure (F = 6.75, *p* < 0.001, ɳ^2^ = 0.02), QRS duration (F = 2.73, *p* = 0.035, ɳ^2^ < 0.01), QTc (F = 6.31, *p* < 0.001, ɳ^2^ = 0.01), P axis (F = 4.77, *p* = 0.002, ɳ^2^ = 0.01), RR interval (F = 28.84, *p* < 0.001, ɳ^2^ = 0.06), and PP interval (F = 15.43, *p* < 0.001, ɳ^2^ = 0.03). Compared to their respective values at PRE, heart rate was decreased at 60 minP on Visit 2 (*p* = 0.018) and Visit 3 (*p* < 0.001), while RR interval and PP interval were both elevated (*p* < 0.001) at 60 minP on Visit 2 and Visit 3; the RR interval was also elevated from Visit 1 at PRE on Visit 2 (*p* = 0.010). QT_C_ (*p* = 0.013) and P axis (*p* = 0.010) were depressed from PRE on Visit 2 at 60 minP only. A trend for decreased systolic blood pressure was noted on Visit 3 PRE compared to Visit 1 (*p* = 0.082). However, neither sex nor group affected these responses. Data collapsed across groups for changes in heart rate and blood pressure are illustrated in Figure 2, while collapsed data derived from the electrocardiogram are presented in Table 2.

Although a group × sex × time interaction was observed for P duration (F = 1.72, *p* = 0.044, ɳ^2^ = 0.02), the observed differences were between sexes at different time points. Specifically, P duration for women consuming 100 mg of Dynamine® was less on Visit 1 (*p* = 0.028) and at 60 minP on Visit 2 (*p* = 0.033) compared to men consuming 100 mg of DYM + 50 mg of TCR at 60 minP on Visit 3. Likewise, that for women consuming 150 mg of DYM was less at 60 minP on Visit 2 compared to men consuming 100 mg of DYM + 50 mg of TCR at 60 minP on Visit 3 (*p* = 0.021). Otherwise, no differences were noted between sexes and groups in the P duration response compared to PRE values. 

### 3.2. Blood Biomarkers

Regardless of group and sex, significant main effects for time were observed for markers analyzed within the complete blood count. Specifically, increased mean corpuscular volume (F = 12.53, *p* < 0.001, ɳ^2^ = 0.01), mean corpuscular hemoglobin (F = 5.81, *p* = 0.018, ɳ^2^ < 0.01), basophils (F = 7.93, *p* = 0.006, ɳ^2^ = 0.03), and absolute eosinophils (F = 6.98, *p* = 0.010, ɳ^2^ = 0.01) were found on Visit 3. Although a group × time interaction was observed for platelets (F = 2.75, *p* = 0.032, ɳ^2^ = 0.01), post hoc analysis did not reveal any significant group differences. Group × sex × time interactions were also observed for mean corpuscular hemoglobin concentration (F = 3.15, *p* = 0.017, ɳ^2^ = 0.02), red cell distribution width (F = 2.53, *p* = 0.045, ɳ^2^ = 0.01), and absolute lymphocytes (F = 2.89, *p* = 0.026, ɳ^2^ = 0.02). However, post hoc analysis only revealed specific differences in mean corpuscular hemoglobin concentrations where values in men consuming the placebo (34.1 ± 0.9 g/dL) were greater than those found in women consuming 100 mg of DYM (32.9 ± 0.8 g/dL, *p* = 0.028) and 150 mg of DYM + 25 mg of TCR (32.8 ± 1.0 g/dL, *p* = 0.011) on Visit 3. No other specific differences were observed. Group and collapsed complete blood count comparisons between Visits 2 and 3 are presented in Table 3 and Table 4.

Regardless of group and sex, significant main time effects were observed for markers analyzed within the complete metabolic panel. Specifically, increased creatinine (F = 8.91, *p* = 0.004, ɳ^2^ < 0.01) and decreased estimated glomerular filtration rate (F = 4.48, *p* = 0.037, ɳ^2^ < 0.01), chloride (F = 4.87, p = 0.030, ɳ^2^ = 0.01), carbon dioxide (F = 5.29, *p* = 0.023, ɳ^2^ = 0.01), bilirubin (F = 5.03, *p* = 0.027, ɳ^2^ < 0.01), and alanine aminotransferase (F = 4.21, *p* = 0.043, ɳ^2^ < 0.01) were noted on Visit 3. Additionally, group x time interactions were seen for blood urea nitrogen (F = 3.30, *p* = 0.014, ɳ^2^ = 0.01), total globulins (F = 3.34, *p* = 0.013, ɳ^2^ = 0.013), alanine aminotransferase (F = 2.82, *p* = 0.028, ɳ^2^ < 0.01), and total proteins (F = 2.61, *p* = 0.040, ɳ^2^ = 0.01), but post hoc analysis only revealed that blood urea nitrogen was greater (*p* = 0.014) in participants consuming 100 mg of DYM than those consuming 100 mg of DYM + 50 mg of TCR on Visit 3. No other differences were observed. Group and collapsed complete metabolic panel comparisons between Visits 2 and 3 are presented in Table 5 and Table 6.

Regardless of group and sex, a significant main effect was found for increased high-density lipoproteins (F = 10.10, *p* = 0.002, ɳ^2^ < 0.01) on Visit 3. Group × time interactions were also seen for triglycerides (F = 2.65, *p* = 0.037, ɳ^2^ = 0.01) and high-density lipoproteins (F = 2.48, *p* = 0.048, ɳ^2^ < 0.01), but post hoc analyses revealed only a trend for increased (*p* = 0.052) high-density lipoproteins for participants consuming 150 mg of Dynamine® on Visit 3 compared to Visit 2. No other differences were observed. Group and collapsed lipid panel comparisons between Visits 2 and 3 are presented in Table 7.

### 3.3. Dietary Tracking

No significant differences in diet were found carbohydrate, fat, protein, or total calories from week one to week four within groups (Table 8). 

## 4. Discussion

To the best of our knowledge, no study previously examined human oral consumption of DYM alone, nor its combination with TCR, and research on TCR alone in humans is somewhat limited. Given their hypothesized similarities with caffeine and potential for reduced habituation and physiological side effects [11,12], DYM and TCR represent intriguing neuro-energetic alternatives. In animal models, TCR [21,32] and DYM [25] alone were determined to be safe. The results of the present study support the hypothesis that 28 days of supplementation does not cause abnormal changes in resting cardiovascular parameters (i.e., heart rate, blood pressure, and electrical conductivity of the heart) or standard hematological safety markers. These findings are in reference to a sample of relatively healthy, young men and women (i.e., similar to the participants in the present study). 

### 4.1. Cardiovascular Function

Resting heart rate is a clinical parameter indicative of health that is easily assessed. Normal values are typically classified between 60 and 100 bpm, and it is not uncommon for values as low as ~30 bpm to be recorded in individuals in excellent physical condition. Moreover, it was found that women often have higher resting heart rates than men [33]. Results from our investigation indicate that, compared to their values at PRE for each respective visit, heart rate was decreased at 60 minP on Visit 2 and Visit 3, regardless of group and sex. Although a negligible decrease in resting HR from PRE was seen on Visit 2 and Visit 3, as evidenced by a less than small effect size, values similarly remained within healthy parameters (i.e., 60–100 bpm).

To date this is the first investigation to examine the impact of DYM on resting heart rate, while a few investigations examined TCR. Li et al. [34] examined the effects of TCR (30 mg/kg) on resting heart rate of hypertensive and normotensive rats at baseline, as well as every 30 min up to 180 min post TCR administration. No changes in resting heart rate were found. Likewise, TCR supplemented at doses of 200 mg and 300 mg by male and female human subjects for eight weeks did not negatively affect resting heart rate [17]. Similar null results were found by Ziegenfuss et al. [14], whose team investigated the acute influence of a 200-mg dose of TCR on heart rate before, as well as 30-minP, 60-minP, 90-minP, 120-minP, 150-minP, and 180-minP supplementation, compared to placebo. Moreover, another study by Kuhman and colleagues [15] compared the effects of men and women’s acute consumption (baseline, and 1 h, 2 h, 3 h, and 4 h post supplementation) of a multi-ingredient dietary supplement containing TCR and caffeine (150 mg) (product name = TheaTrim) to caffeine alone and placebo, and they also found no differences in resting heart rate response across groups. Similar results were also reported by He and colleagues in healthy adults [16]. High resting heart rate is now considered an important constituent for increasing the chance of mortality, and this relationship is independent of age, sex, or blood pressure in adults [35]. As DYM and TCR may become a dietary supplement consumed on a regular basis, like caffeine is for many, establishing its negligible impact on resting heart rate is important for maintaining health. 

Resting blood pressure is also an important indicator of cardiovascular health, with chronic elevated blood pressure (i.e., hypertension) acknowledged to increase cardiovascular disease risk. Hypertension was recently defined as a systolic blood pressure of 130 or greater and a diastolic blood pressure of 80 or greater [36]. Caffeine consumption is well known to acutely increase blood pressure, likely through antagonism of the alpha-adrenergic receptors 1 and 2 (or α1 and α2) throughout the body [37], and its ability to enhance vascular resistance raises questions regarding its impact on the development of hypertension [37]. Therefore, the influence of TCR and DYM on blood pressure is of interest, due to their structural similarities to caffeine. One investigation in rats examined the impact of TCR on alpha-adrenergic receptors and found that pre-treatment caused adenosine receptor antagonism, as demonstrated by TCR’s ability to counteract the motor depression induced by adenosine receptor agonists (i.e., CPA and CGS-21680) [24]. Based on this evidence, one may expect TCR, and possibly DYM, to influence systolic and diastolic blood pressure like caffeine. However, our data do not support this hypothesis as we found no significant acute or chronic effects of DYM alone or in combination with TCR on systolic or diastolic blood pressure. Rather, our findings appear to corroborate those of four other studies examining TCR in humans [14,15,17,19], and one in rats [34], which reported no observable negative effects on blood pressure [14,17], even when TCR was combined with caffeine [15,19]. 

In addition to heart rate and blood pressure, a resting ECG assessment was collected prior to supplementation and at 60 minP on each visit to further examine the acute cardiovascular safety of DYM and its combination with TCR. Again, no abnormal changes in electrical activity of the heart were observed for any experimental group across visits. The International Conference for Harmonization E14 highlighted the importance of thorough QT/QTc assessments of new non-antiarrhythmic drugs [38]. QT prolongation is a surrogate marker for delayed myocardial repolarization and is associated with a higher risk of cardiovascular events (i.e., torsades de pointes, an uncommon form of polymorphic ventricular tachycardia) and mortality in not only cardiac patients, but also the general population [39]. While both DYM and TCR are categorized as dietary supplements, their potential stimulatory effects warranted their QTc examination. When groups were combined, ECG QTc assessments indicated a significant average drop of ~4 ms during the 60-minP assessment on Visit 2. This change is within the threshold level of regulatory concern often used within thorough QT/QTc studies (i.e., 5 ms) [38]. Overall, parameters associated with electrical conduction of the heart remained within normal parameters, despite the supplement consumed. 

### 4.2. Blood Biomarkers

Hematological markers associated with health were examined, with no abnormal results found following 28 days of supplementation. Mean values fell within normal clinical reference ranges for all markers associated with CBC, CMP, and the lipid panel, excluding hemoglobin at Visit 3 for the group consuming 150 mg of DYM and calcium for the 100-mg DYM group. Changes were not significant, and we do not suspect DYM was responsible for these concentration fluctuations. Furthermore, collapsed results across groups indicate increased mean corpuscular volume, mean corpuscular hemoglobin, absolute eosinophils, creatinine, estimated glomerular filtration rate, chloride, total carbon dioxide, bilirubin, alanine aminotransferase, and high density lipoprotein (HDL) on Visit 3; however, no values fell outside what was deemed healthy for participants involved in this study and would likely be considered stochastic. Researchers examining clinical hematological data in male and female rats supplemented with DYM for 90 days (0, 75, 112, 150, 187, and 225 mg/kg) and two additional groups of five rats per sex that were administered 0 and 225 mg/kg for further evaluation following a 28-day recovery period found that the majority of the hematological results remained within or marginal to historical control ranges, suggesting no toxicity concerns [25]. In the only human study examining the effect of TCR on clinical blood markers, Taylor et al. [17] reported no concerns related to dosages of 200 and 300 mg over eight weeks. Interestingly, individuals consuming the 300-mg dose of TCR experienced decreases in total cholesterol and low-density lipoproteins, at the four- and eight-week assessments, respectively. While our results did not indicate changes in these lipid markers, collapsed results indicated an increase in high-density lipoproteins. More research should be conducted on DYM and TCR alone and in combination as it relates to cholesterol, as tea (e.g., green tea), which contains alkaloids, was demonstrated to beneficially influence cholesterol profiles [40]. 

## 5. Conclusions, Limitations, and Future Directions 

This is the first study to examine DYM alone or in combination with TCR in humans. Our findings indicate that once-daily supplementation with DYM alone (100 mg and 150 mg) or in combination with TCR (DYM 100 mg + TCR 50 mg or DYM 150 mg + 25 mg TCR) consumed by apparently healthy, young men and women does not negatively impact health, as measured by cardiovascular function and a comprehensive hematological panel. 

As with all studies, there are limitations to our research which we openly acknowledge. Firstly, our safety assessments of health were limited to tests of cardiovascular function (heart rate, blood pressure, ECG) and routine blood work (complete blood count, comprehensive metabolic panel, and lipid panel). Secondly, our subject sample included only younger men and women; future studies may consider examining older adults, as both younger and older individuals may be interested in using TCR and DYM. Thirdly, our participant inclusion criteria ranged from sedentary to recreationally active individuals. As exercise may impact caffeine kinetics, including potentiation of its action, reduction in the extent of its effects, and acceleration of its elimination, there is the possibility that participants who engaged in more exercise than others (i.e., sedentary) experienced different rates of metabolism and excretion of their designated supplement (due to the similar structures to caffeine), which may have impacted the results. Finally, we recommend reproducing our study design with larger sample sizes to more thoroughly interrogate the effects of DYM and TCR on health. In addition, future studies should examine the impact of DYM alone or in combination with TCR, and possibly caffeine, on cognitive function and exercise performance, as these ingredients are currently being included in pre-workout and nootropic supplements.

## Figures and Tables

**Table 1:** Baseline participant descriptions. Values are means ± SD. M—male; F—female.

	Sample Size	Sex	Age (year)	Height (cm)	Body Mass (kg)	Body Fat (%)	Resting Heart Rate (bpm)	Resting Systolic Blood Pressure (mmHg)	Resting Diastolic Blood Pressure (mmHg)
100 mg DYM	12	M	21.7 ± 1.3	174.5 ± 7.8	78.2 ± 11.1	16.7 ± 6.3	63.3 ± 7.5	123.0 ± 7.0	75.8 ± 8.4
	13	F	23.4 ± 2.7	162.6 ± 4.4	62.7 ± 10.9	30.6 ± 6.7	66.0 ± 11.3	110.2 ± 3.8	67.0 ± 7.6
100 mg DYM + 50 mg TCR	12	M	23.5 ± 3.0	175.1 ± 6.7	83.3 ± 10.3	24.2 ± 7.0	66.9 ± 10.9	120.0 ± 6.4	74.1 ± 10.2
	13	F	24.0 ± 6.5	163.3 ± 5.1	64.4 ± 14.1	31.7 ± 7.5	61.3 ± 7.6	107.5 ± 4.9	64.6 ± 5.2
150 mg DYM +25 mg TCR	12	M	24.2 ± 3.3	175.7 ± 3.5	78.1 ± 5.6	19.5 ± 2.9	63.6 ± 8.8	117.6 ± 5.8	71.5 ± 9.1
	13	F	23.1 ± 2.6	164.4 ± 5.1	62.7 ± 8.0	30.7 ± 7.1	73.8 ± 10.1	111.0 ± 6.8	71.0 ± 5.4
150 mg DYM	12	M	22.8 ± 2.6	178.3 ± 7.3	80.6 ± 8.2	19.8 ± 5.6	61.1 ± 10.7	116.3 ± 7.8	74.4 ± 8.7
	13	F	21.8 ± 1.8	162.7 ± 8.6	63.0 ± 15.0	27.7 ± 9.4	73.7 ± 11.2	111.2 ± 8.0	68.1 ± 6.1
Placebo (maltodextrin)	12	M	22.8 ± 3.3	117.2 ± 7.7	82.5 ± 14.0	23.8 ± 7.8	66.9 ± 3.9	117.7 ± 7.1	76.8 ± 7.0
	13	F	22.9 ± 3.8	166.5 ± 4.7	70.9 ± 11.9	31.1 ± 6.9	68.8 ± 10.1	110.0 ± 5.3	67.3 ± 7.6

**Table 2:** Collapsed (*n* = 125; group and sex) data for group changes in electrocardiogram measures. *n* = 25 participants per group.

	Visit 1	Visit 2		Visit 3	
	PRE	PRE	60 minP	PRE	60 minP
PR Interval (ms)	145 ± 17	146 ± 17	147 ± 17	145 ± 16	149 ± 34
QRS Duration (ms)	88.7 ± 9	89.7 ± 9	89.8 ± 8.8	89.6 ± 9.1	89.2 ± 8.9
QT_C_ (ms)	412 ± 23	410 ± 23	406 ± 24 *	412 ± 23	409 ± 24
P Axis (◦)	56.4 ± 19.1	56.5 ± 19.1	51.9 ± 22.3 *	53.9 ± 20.9	51.5 ± 22.2
R Axis (◦)	75.7 ± 15.3	75.5 ± 17.3	74.1 ± 17.5	75.4 ± 17.4	75.1 ± 17.4
T Axis (◦)	51.8 ± 14	53 ± 14.3	53.6 ± 17.6	52.6 ± 13.6	52.4 ± 13.3
P Duration (ms)	94.5 ± 10.2	94.2 ± 10.1	93.3 ± 11.3	93.4 ± 10	94.4 ± 10.2
RR Interval (ms)	928 ± 158	961 ± 157 #	1030 ± 166 *	950 ± 185	1024 ± 176 *
PP Interval (ms)	935 ± 214	959 ± 161	1022 ± 173 *	948 ± 188	1010 ± 167 *

* Significantly (*p* < 0.05) different from PRE on respective visit. # Significantly (*p* < 0.05) different from Visit 1. minP = minutes post PRE; PR Interval = time from the onset of the P wave to the start of the QRS complex; QRS duration = ventricular depolarization; QT = time duration between the onset of the QRS complex and the end of the T wave (ventricular depolarization to complete repolarization); c = corrected for heart rate; P duration = time of P wave (atrial depolarization); RR interval = time elapsed between successive R waves; PP interval = time elapsed between successive P waves; axis represents overall electrical activity of the heart.

**Table 3:** Group (*n* = 25 per group) and average complete blood count comparisons between Visits 2 and 3.

		100 mg of DYM	100 mg of DYM + 50 mg of TCR	150 mg of DYM + 25 mg of TCR	150 mg of DYM	Placebo	Average
White Blood Cells (× 10^3^/µL)	Visit 2	5.37 ± 1.26	6.20 ± 1.62	5.78 ± 1.26	5.76 ± 1.39	5.50 ± 1.58	5.71 ± 1.43
	Visit 3	5.68 ± 1.62	5.92 ± 1.97	5.71 ± 1.28	5.56 ± 1.30	5.53 ± 1.29	5.68 ± 1.49
Red Blood Cells (× 10^6^/µL)	Visit 2	4.83 ± 0.41	4.84 ± 0.41	4.64 ± 0.40	4.65 ± 0.33	4.83 ± 0.57	4.76 ± 0.43
	Visit 3	4.83 ± 0.39	4.82 ± 0.47	4.61 ± 0.40	4.67 ± 0.26	4.84 ± 0.53	4.76 ± 0.42
Hemoglobin (g/dL)	Visit 2	14.6 ± 1.0	14.4 ± 1.0	13.6 ± 1.0	14.1 ± 0.9	14.2 ± 1.2	14.1 ± 1.0
	Visit 3	14.6 ± 1.0	14.3 ± 1.1	13.7 ± 1.1	19.5 ± 25.1	14.2 ± 1.2	15.2 ± 11.1
Hematocrit (%)	Visit 2	43.4 ± 2.9	43.7 ± 11.7	40.3 ± 2.2	42 ± 3	42.3 ± 3.4	42.3 ± 5.6
	Visit 3	44.1 ± 2.9	42.7 ± 3.0	40.9 ± 2.6	41.4 ± 3.4	42.7 ± 3.1	42.3 ± 3.2
Mean Corpuscular Volume (fL)	Visit 2	89.7 ± 3.6	88.5 ± 3.6	88.4 ± 4.8	88.7 ± 4.7	88.0 ± 5.0	88.6 ± 4.4
	Visit 3	91.0 ± 3.6	88.9 ± 3.2	88.8 ± 4.3	89.3 ± 5.5	88.6 ± 6.2	89.3 ± 4.7*
Mean Corpuscular Hemoglobin (pg)	Visit 2	29.9 ± 1.5	29.7 ± 1.0	29.9 ± 1.8	30.1 ± 1.9	29.4 ± 1.8	29.8 ± 1.6
	Visit 3	30.2 ± 1.1	29.7 ± 1.1	30.1 ± 1.6	30.5 ± 1.9	29.6 ± 1.9	30.0 ± 1.6 *
Mean Corpuscular Hemoglobin Concentration (g/dL)	Visit 2	33.4 ± 0.8	33.6 ± 0.8	33.3 ± 1.1	33.5 ± 0.8	33.6 ± 0.9	33.5 ± 0.9
	Visit 3	33.0 ± 0.8	33.5 ± 0.5	33.4 ± 1.1	33.4 ± 0.9	33.7 ± 0.9	33.4 ± 0.9
Red Cell Distribution Width (%)	Visit 2	13.4 ± 0.7	13.2 ± 0.5	13.2 ± 0.9	13.2 ± 0.6	13.4 ± 0.6	13.3 ± 0.6
	Visit 3	13.3 ± 0.5	13.3 ± 0.5	13.3 ± 0.7	13.3 ± 0.6	13.4 ± 0.6	13.3 ± 0.6
Platelets (× 10^3^/µL)	Visit 2	236 ± 55	265 ± 48	254 ± 42	240 ± 49	255 ± 55	250 ± 50
	Visit 3	220 ± 60	246 ± 57	256 ± 41	247 ± 50	266 ± 61	247 ± 56

* Significantly (*p* < 0.05) different from Visit 2.

**Table 4:** Group (*n* = 25 per group) and average complete blood count comparisons between Visits 2 and 3.

		100 mg of DYM	100 mg of DYM + 50 mg of TCR	150 mg of DYM + 25 mg of TCR	150 mg of DYM	Placebo	Average
Absolute Neutrophils (× 10^3^/µL)	Visit 2	2.68 ± 1.04	3.06 ± 1.47	2.82 ± 0.85	2.77 ± 1.24	2.56 ± 1.30	2.77 ± 1.18
	Visit 3	2.78 ± 1.28	2.85 ± 1.58	2.82 ± 0.85	2.58 ± 1.12	2.60 ± 0.90	2.72 ± 1.15
Absolute Lymphocytes (× 10^3^/µL)	Visit 2	2.09 ± 0.33	2.31 ± 0.44	2.30 ± 0.50	2.13 ± 0.51	2.07 ± 0.51	2.18 ± 0.47
	Visit 3	2.25 ± 0.50	2.31 ± 0.72	2.24 ± 0.51	2.20 ± 0.48	2.20 ± 0.60	2.24 ± 0.56
Absolute Monocytes (× 10^3^/µL)	Visit 2	0.463 ± 0.124	0.519 ± 0.172	0.432 ± 0.149	0.396 ± 0.112	0.475 ± 0.165	0.455 ± 0.149
	Visit 3	0.445 ± 0.122	0.491 ± 0.216	0.470 ± 0.122	0.414 ± 0.159	0.467 ± 0.134	0.458 ± 0.153
Absolute Eosinophils (× 10^3^/µL)	Visit 2	0.183 ± 0.169	0.162 ± 0.102	0.144 ± 0.112	0.138 ± 0.077	0.175 ± 0.099	0.160 ± 0.116
	Visit 3	0.186 ± 0.146	0.191 ± 0.177	0.157 ± 0.112	0.181 ± 0.112	0.192 ± 0.132	0.181 ± 0.136 *
Absolute Basophils (× 10^3^/µL)	Visit 2	0.017 ± 0.038	0.014 ± 0.036	0.012 ± 0.033	<0.010	<0.010	0.008 ± 0.028
	Visit 3	0.009 ± 0.029	0.009 ± 0.029	0.027 ± 0.046	0.014 ± 0.036	0.008 ± 0.028	0.014 ± 0.034
Immature Granulocytes (× 10^3^/µL)	Visit 2	0.208 ± 0.415	0.429 ± 0.978	0.080 ± 0.277	0.261 ± 0.689	0.333 ± 0.637	0.256 ± 0.632
	Visit 3	0.318 ± 0.477	0.364 ± 0.79	<0.010	0.048 ± 0.218	0.208 ± 0.658	0.188 ± 0.529
Absolute Immature Granulocytes (× 10^3^/µL)	Visit 2	0.017 ± 0.038	0.029 ± 0.072	0.004 ± 0.020	0.009 ± 0.029	0.025 ± 0.044	0.016 ± 0.043
	Visit 3	0.014 ± 0.035	0.014 ± 0.035	<0.010	0.010 ± 0.030	0.013 ± 0.034	0.010 ± 0.030

* Significantly (*p* < 0.05) different from Visit 2.

**Table 5:** Group (*n* = 25 per group) and average complete metabolic panel comparisons between Visits 2 and 3.

		100 mg of DYM	100 mg of DYM + 50 mg of TCR	150 mg of DYM + 25 mg of TCR	150 mg of DYM	Placebo	Average
Glucose (mg/dL)	Visit 2	87.5 ± 7.2	89.1 ± 7.4	90.2 ± 5.2	91.2 ± 9.4	91.0 ± 8.5	89.8 ± 7.7
	Visit 3	87.2 ± 8.5	88.2 ± 9.1	88.9 ± 6.4	91.9 ± 7.6	89.9 ± 8.8	89.2 ± 8.2
Blood Urea Nitrogen (mg/dL)	Visit 2	15.2 ± 4.1	14.0 ± 4.0	14.0 ± 4.6	14.2 ± 4.1	14.0 ± 3.3	14.3 ± 4.0
	Visit 3	16.5 ± 4.7	12.6 ± 3.7#	14.3 ± 4.9	14.0 ± 3.9	14.1 ± 2.8	14.3 ± 4.2
Creatinine (mg/dL)	Visit 2	0.984 ± 0.220	0.955 ± 0.161	0.870 ± 0.191	0.947 ± 0.246	0.908 ± 0.172	0.932 ± 0.201
	Visit 3	1.003 ± 0.210	0.999 ± 0.177	0.889 ± 0.198	0.981 ± 0.230	0.914 ± 0.172	0.957 ± 0.200 *
Estimated Glomerular Filtration Rate (mL/min/1.73)	Visit 2	96.6 ± 17.8	94.1 ± 26.3	107.8 ± 16.7	93.3 ± 29.0	104.0 ± 14.9	99.3 ± 22.0
	Visit 3	84.1 ± 31.0	92.0 ± 15.4	105.7 ± 15.4	91.6 ± 26.7	103.8 ± 16	95.5 ± 22.9 *
Blood Urea Nitrogen–Creatinine Ratio	Visit 2	16.1 ± 5.0	14.8 ± 4.4	16.0 ± 4.2	14.9 ± 5.4	15.3 ± 3.3	15.4 ± 4.5
	Visit 3	16.7 ± 4.2	12.9 ± 4.1	16.0 ± 4.0	14.7 ± 4.1	15.5 ± 3.1	15.1 ± 4.1
Sodium (mmol/L)	Visit 2	141 ± 2	140 ± 2	140 ± 2	141 ± 2	140 ± 2	141 ± 2
	Visit 3	141 ± 1	140 ± 2	140 ± 2	141 ± 2	141 ± 2	141 ± 2
Potassium (mmol/L)	Visit 2	4.80 ± 0.42	4.62 ± 0.48	4.46 ± 0.44	4.98 ± 1.91	4.35 ± 0.32	4.64 ± 0.95
	Visit 3	4.61 ± 0.52	4.63 ± 0.83	4.41 ± 0.29	4.64 ± 0.70	4.60 ± 0.42	4.58 ± 0.59
Chloride (mmol/L)	Visit 2	102 ± 3	101 ± 4	102 ± 2	103 ± 2	102 ± 2	102 ± 3
	Visit 3	103 ± 2	103 ± 2	102 ± 2	103 ± 3	103 ± 2	103 ± 2 *
Total Carbon Dioxide (mmol/L)	Visit 2	24.3 ± 2.3	24.3 ± 2.0	23.2 ± 2.5	23.4 ± 2.2	23.9 ± 2.2	23.8 ± 2.2
	Visit 3	23.2 ± 2.1	23.3 ± 1.7	22.2 ± 2.3	23.2 ± 2.6	24.3 ± 2.3	23.3 ± 2.3 *
Calcium (mg/dL)	Visit 2	9.64 ± 0.27	9.53 ± 0.31	9.47 ± 0.37	9.32 ± 0.45	9.53 ± 0.34	9.50 ± 0.37
	Visit 3	16.77 ± 23.90	9.40 ± 0.34	9.44 ± 0.29	9.44 ± 0.33	9.50 ± 0.38	10.87 ± 10.77

* Significantly (*p* < 0.05) different from Visit 2. # Significantly (*p* < 0.05) different from 100 mg of Dynamine on Visit 3.

**Table 6:** Group (*n* = 25 per group) and average complete metabolic panel comparisons between Visits 2 and 3.

		100 mg of DYM	100 mg of DYM + 50 mg of TCR	150 mg of DYM + 25 mg of TCR	150 mg of DYM	Placebo	Average
Total Protein (g/dL)	Visit 2	7.18 ± 0.34	7.26 ± 0.52	7.06 ± 0.53	6.92 ± 0.72	7.18 ± 0.25	7.12 ± 0.51
	Visit 3	7.07 ± 0.31	7.17 ± 0.51	7.08 ± 0.36	7.11 ± 0.76	7.18 ± 0.39	7.12 ± 0.49
Albumin (A) (g/dL)	Visit 2	4.67 ± 0.26	4.61 ± 0.33	4.60 ± 0.42	4.56 ± 0.30	4.66 ± 0.32	4.62 ± 0.33
	Visit 3	4.65 ± 0.26	4.53 ± 0.31	4.54 ± 0.38	4.62 ± 0.42	4.64 ± 0.31	4.59 ± 0.34
Total Globulin (G) (g/dL)	Visit 2	2.47 ± 0.19	2.65 ± 0.40	2.58 ± 0.43	2.59 ± 0.42	2.58 ± 0.35	2.57 ± 0.37
	Visit 3	2.43 ± 0.23	2.52 ± 0.42	2.67 ± 0.38	2.67 ± 0.38	2.58 ± 0.36	2.57 ± 0.37
A/G Ratio	Visit 2	1.90 ± 0.19	1.78 ± 0.31	1.83 ± 0.35	1.81 ± 0.31	1.84 ± 0.32	1.83 ± 0.30
	Visit 3	1.94 ± 0.24	1.84 ± 0.3	1.73 ± 0.28	1.76 ± 0.31	1.84 ± 0.29	1.82 ± 0.29
Bilirubin, Total (mg/dL)	Visit 2	0.529 ± 0.229	0.496 ± 0.272	0.504 ± 0.348	0.424 ± 0.230	0.496 ± 0.321	0.489 ± 0.282
	Visit 3	0.448 ± 0.250	0.468 ± 0.281	0.483 ± 0.248	0.391 ± 0.095	0.454 ± 0.213	0.449 ± 0.226 *
Alkaline Phosphatase (IU/L)	Visit 2	61.8 ± 15.6	66.9 ± 23.1	66.4 ± 21.3	62.5 ± 15.8	68.4 ± 18.2	65.2 ± 18.9
	Visit 3	60.3 ± 15.5	65.5 ± 20.8	67.6 ± 21.2	62.8 ± 15.6	71.4 ± 24.4	65.6 ± 19.9
Aspartate Aminotransferase (IU/L)	Visit 2	30.9 ± 22.8	23.4 ± 10.0	21.7 ± 7.2	23.2 ± 8.2	24.6 ± 8.9	24.7 ± 12.9
	Visit 3	28.9 ± 17.7	24.7 ± 10.1	23.2 ± 8.9	24.6 ± 11.6	22.0 ± 7.1	24.7 ± 11.6
Alanine Aminotransferase (IU/L)	Visit 2	23.8 ± 11.7	22.5 ± 22.7	19.2 ± 9.7	14.8 ± 6.4	21.3 ± 10.6	20.3 ± 13.6
	Visit 3	19.6 ± 7.9	22.4 ± 18.0	19.7 ± 10	15.7 ± 6.7	18.8 ± 9.6	19.3 ± 11.3 *

* Significantly (*p* < 0.05) different from Visit 2.

**Table 7:** Group (*n* = 25 per group) and average lipid panel comparisons between Visits 2 and 3.

		100 mg of DYM	100 mg of DYM + 50 mg of TCR	150 mg of DYM + 25 mg of TCR	150 mg of DYM	Placebo	Average
Total Cholesterol (mg/dL)	Visit 2	158 ± 26	167 ± 30	155 ± 34	145 ± 22	175 ± 22	160 ± 28
	Visit 3	160 ± 35	166 ± 24	157 ± 34	148 ± 23	167 ± 34	160 ± 31
Triglycerides (mg/dL)	Visit 2	69.8 ± 29.7	86.3 ± 39.6	84.9 ± 32.2	84.3 ± 39.5	96.0 ± 60.6	84.3 ± 41.8
	Visit 3	75.3 ± 31.9	77.8 ± 35.5	91.1 ± 41.3	83.4 ± 30.5	82.2 ± 44.1	81.9 ± 36.8
High-Density Lipoproteins (mg/dL)	Visit 2	63.8 ± 13.9	56.7 ± 13.7	59.3 ± 12.4	54.7 ± 12.3	58.2 ± 17.4	58.5 ± 14.1
	Visit 3	63.2 ± 14.0	60.0 ± 16.1	61.1 ± 15.6	59.5 ± 14.2	57.4 ± 22.2	60.2 ± 16.5 *
Very-Low-Density Lipoproteins (mg/dL)	Visit 2	13.9 ± 6.0	17.2 ± 7.9	15.4 ± 5.5	15.8 ± 7.9	18.0 ± 11.6	16.0 ± 8.0
	Visit 3	15.0 ± 6.6	15.8 ± 7.4	16.8 ± 7.8	16.3 ± 6.1	17.2 ± 9.4	16.2 ± 7.4
Low-Density Lipoproteins (mg/dL)	Visit 2	80.8 ± 21.0	93.8 ± 23.9	80.0 ± 28.5	74.9 ± 19.7	98.6 ± 22.4	85.6 ± 24.6
	Visit 3	81.6 ± 26.9	90.2 ± 24.2	79.5 ± 27.3	72.3 ± 20.1	96.4 ± 24.4	84.2 ± 25.7

* Significantly (*p* < 0.05) different from Visit 2.

**Table 8:** Macronutrient intakes and total calories at week one and week four for all participants. Values are mean ± SD.

	Sample Size	Sex		Carbohydrates (g)	Fat (g)	Protein (g)	Total Calories	*p*-Value
100 mg of DYM	12	M	Week 1	202 ± 43	72 ± 20	177 ± 27	2166 ± 323	0.17
			Week 4	206 ± 39	64 ± 34	166 ± 34	2060 ± 311	
	13	F	Week 1	174 ± 52	61 ± 26	98 ± 34	1637 ± 409	0.72
			Week 4	188 ± 46	63 ± 25	90 ± 25	1681 ± 356	
100 mg of DYM + 50 mg of TCR	12	M	Week 1	231 ± 30	67 ± 20	118 ± 33	2002 ± 253	0.13
			Week 4	229 ± 33	75 ± 26	126 ± 27	2091 ± 268	
	13	F	Week 1	181 ± 31	43 ± 12	95 ± 22	1488 ± 221	0.53
			Week 4	176 ± 29	52 ± 19	89 ± 22	1528 ± 174	
150 mg of DYM +25 mg of TCR	12	M	Week 1	238 ± 57	74 ± 34	147 ± 51	2172 ± 330	0.48
			Week 4	253 ± 52	70 ± 34	150 ± 62	2244 ± 517	
	13	F	Week 1	186 ± 79	58 ± 13	90 ± 28	1627 ± 337	0.75
			Week 4	194 ± 68	54 ± 14	96 ± 22	1652 ± 307	
150 mg of DYM	12	M	Week 1	198 ± 214	72 ± 24	161 ± 48	2087 ± 345	0.66
			Week 4	214 ± 43	65 ± 20	149 ± 49	2037 ± 388	
	13	F	Week 1	166 ± 46	56 ± 13	83 ± 27	1501 ± 232	0.12
			Week 4	171 ± 58	62 ± 16	89 ± 21	1600 ± 306	
Placebo (maltodextrin)	12	M	Week 1	170 ± 56	85 ± 20	158 ± 65	2078 ± 405	0.73
			Week 4	185 ± 42	72 ± 23	161 ± 60	2028 ± 212	
	13	F	Week 1	213 ± 85	56 ± 26	89 ± 32	1711 ± 425	0.15
			Week 4	186 ± 98	57 ± 21	84 ± 37	1598 ± 434	
